# Changes in human mandibular shape during the Terminal Pleistocene-Holocene Levant

**DOI:** 10.1038/s41598-019-45279-9

**Published:** 2019-06-19

**Authors:** Ariel Pokhojaev, Hadas Leah Levine, Tatiana Sella-Tunis, Rachel Sarig, Hila May

**Affiliations:** 1 Department of Anatomy and Anthropology, Sackler Faculty of Medicine, Tel Aviv University, Ramat Aviv, Tel Aviv 69978 Israel; 2 Shmunis Family Anthropology Institute, Dan David Center for Human Evolution and Biohistory Research, Sackler Faculty of Medicine, Steinhardt Natural History Museum, Tel Aviv University, Ramat Aviv, Tel Aviv 6997801 Israel; 3 Departments of Orthodontics and Oral Biology, The Maurice and Gabriela Goldschleger School of Dental Medicine, Sackler Faculty of Medicine, Tel Aviv University, Ramat Aviv, Tel Aviv 69978 Israel

**Keywords:** Biological anthropology, Oral anatomy

## Abstract

The transition to food production, exploitation of ‘secondary’ products (e.g., milk), and advances in cookware technology have affected all aspects of human life. The aim of the present study was to follow changes in mandibular form and shape throughout the terminal Pleistocene-Holocene Levant. The hemimandibles of four populations were included in this study: Natufian hunter-gatherers (n = 10), Pre-pottery Neolithic early farmers (n = 6), Chalcolithic farmers (n = 9), Roman-Byzantine (n = 16), and modern (n = 63) populations. A surface mesh of each mandible was reconstructed from CT or surface scans. Changes in mandibular form and shape were studied using the Procrustes-based geometric morphometrics method. Univariate and multivariate analyses were carried out to examine differences in size and shape between the studied populations. Our results reveal considerable temporal changes in mandibular shape throughout the Holocene Levant, mainly between the pre-agricultural population (the Natufian) and the succeeding ones, and between the post-industrial (the Modern) and the pre-industrial populations. A tendency for a reduction in mandibular size was identified between the pre-agricultural population and the farmers. Most regions of the mandible underwent shape changes. In conclusion, substantial changes in mandibular shape occurred throughout the Holocene Levant, especially following the agricultural revolution. These changes can be explained by the “masticatory-functional hypothesis”.

## Introduction

The impact of dietary changes on mandibular morphology (e.g.^1–4^) and their implications in common oral disorders^1,5,6^ have been discussed in many studies. Nevertheless, to the best of our knowledge, few studies have systematically followed changes in the shape of the mandible in restricted geographical regions^3,4^. Moreover, none of these studies examined changes during the entire Holocene period (until nowadays). In this study, we aimed to follow temporal changes in the three-dimensional (3D) shape of the mandible throughout the terminal Pleistocene-Holocene Levant using the Procrustes-based geometric morphometrics method.

The Levantine populations shifted to a food-producing economy earlier than most other human populations, during the Pre-pottery Neolithic period (12,175–8,450 cal BP). This shift prompted changes in all aspects of human life: economical, socio-cultural, biological, and technological^4,6–17^. The intensive hunting and gathering that had been widely practiced by the terminal Pleistocene Natufians (14,900–11,750 cal BP) were abandoned in favor of land cultivation as well as plant and animal domestication. Large villages with constructed housing replaced caves and small open-site settlements, and public buildings appeared. Nevertheless, further changes were soon to follow with the introduction of cookware in the Pottery Neolithic period (8,400–6,500 cal BP); food preparation moved into a new phase, enabling lengthy cooking in pots (e.g.^9,10,18–20^). This advancement not only enlarged the variety of edible vegetables and elevated the amount of nutrients and energy that could be obtained from them, it also reduced demands from the masticatory system (in force and time)^21^. A few thousand years later, during the Chalcolithic period (6,500–5,500 cal BP), the consumption of dairy products (the “secondary product revolution”), following the invention of churns, became common^22–24^, expanding the variety of soft foods.

These developments in food preparation techniques resulted in a more processed and refined diet, which requires lower masticatory forces. According to the “masticatory-functional hypothesis”^25^, the reduction in mastication forces should be reflected in mandibular morphology^1,2,26–33^. Various studies have tried to follow changes in mandibular morphology using the time dimension and the changes in dietary habits that followed it. It was demonstrated that in the Levant, as well as in other geographical regions, mandibular size (e.g., body length, ramus width, symphysis height, and coronoid width) was reduced continuously throughout time in accordance with subsistence changes^2,3,6,32,34–40^. It was also suggested that the orientation of the mandible changed over time, which was manifested in an increased mandibular angle and a more vertical inclination of the mandibular body^32,41^. In the Levant, however, most studies have focused mainly on the Natufian and Neolithic populations^4,6,38–40,42^. However, a recent study by May *et al*.^32^ suggested that many of the morphological changes including mandibular body height at the molar region, and the cross-sectional area of the mandibular body and ramus occurred only after the Chalcolithic period (<6,000 years). Moreover, the above-mentioned studies provided limited insight, since they used mainly linear and angular two-dimensional measurements, which are less sensitive in capturing subtle temporal morphological differences compared with shape analysis^43^. Thus, using Geometric morphometric methods may be a better way to examine the association between changes in dietary habits through time and changes in mandibular shape. This approach has already been proven effective in identifying mandibular shape differences between populations that varied in subsistence strategy^1–3,33,44^, and in determining the association between mandibular shape and muscle force^45,46^ or biting performance^33^. The current study was aimed to identify mandibular shape changes in populations of the southern Levant during the last 15,000 years. Three hypotheses were raised: The first, the Pre-agriculture population (the Natufian) will manifest different mandibular form and shape compared with all succeeding populations. The second, the post-industrial population (the modern population) will manifest different mandibular characteristics compared with the historic and prehistoric populations; and the third, the differences in form and shape will increase over time.

## Results

### Outliers

Five modern individuals (two females and three males) were excluded from the analysis. Thus, the sample size included 98 individuals: 10 Natufian, 6 Neolithic, 9 Chalcolithic, 16 Roman/Byzantine, and 57 modern mandibles.

### Form analysis

**Figure 1:**
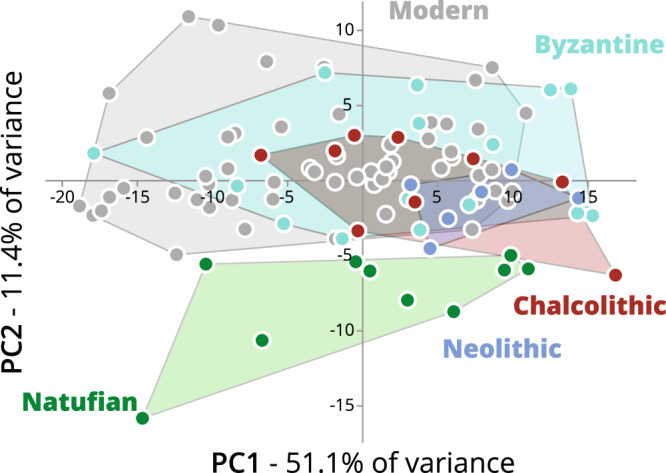
Principal component analysis of hemimandibular form by population (using the set of nine landmarks): Natufian (green), Neolithic (blue), Chalcolithic (brown), Roman/Byzantine (light blue) and modern (gray). The first two principal components (PC) explain 62.5% of the variance.

**Figure 2:**
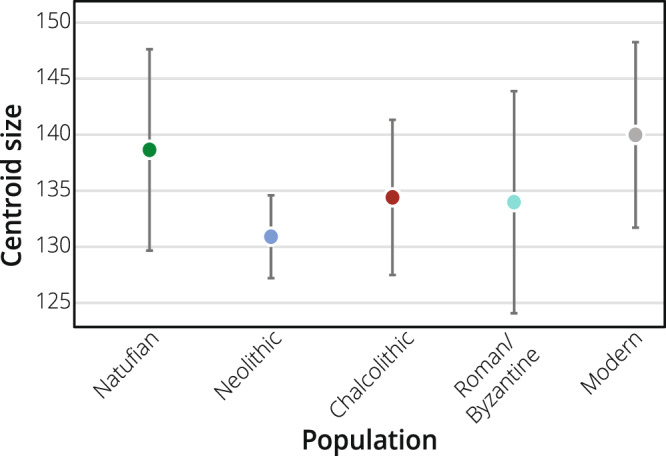
Hemimandibular size (mean and standard deviation) by population: Natufian, Neolithic, Chalcolithic, Roman/Byzantine, and modern. No significant differences in size were found between the studied populations. Statistical results are presented in Supplementary Table S1.

The first two components of the Principal Component Analysis (PCA) of the form (shape + size) of the hemimandibles included in the study explain 62.5% of the variance based on the nine-landmark set (see the Methods section) (Fig. 1). The second PC, explains 11.4% of the variance, differentiates between the Natufian hemimandibles and the other hemimandibles studied. Although among the post-Natufian populations, variance overlaps considerably, the Neolithic hemimandibles fell within the variance of the Chalcolithic hemimandibles, which are concentrated mainly in the first and second quadrates of the coordinate system. The Roman/Byzantine and Modern hemimandibles had larger variances and are also spread in the third and fourth quadrates of the coordinate system (Fig. 1). Following the overlap in form variance between the Neolithic and Chalcolithic hemimandibles, the Pairwise permutation multivariate analysis of variance (PERMANOVA) of Procrustes distances was carried out on four groups (Natufian, Neolithic-Chalcolithic, Roman/Byzantine, and Modern). Form differences almost reach significance between the Natufian and Neolithic/Chalcolithic hemimandibles (*p* = 0.054) and the form of the Modern hemimandible differs significantly from that of the other studied groups (Table 1). Nevertheless, the hemi-mandibular size did not differ significantly between the studied groups, although the Natufian hemimandibles were larger than those of the Neolithic, Chalcolithic, and Roman/Byzantine (hereafter, termed post-Natufian) hemimandibles and were similar to the Modern ones (Fig. 2; Supplementary Table S1). Since no significant differences in form were found between the post-Natufian populations, we combined them for the linear discriminant analysis (LDA). This analysis, after cross-validation (using the Jackknife method), gave ca. a 70% correct classification rate for each group (Table 2).

**Table 1:** Pairwise permutational multivariate analysis of variance (with 1000 permutations) on Procrustes distances between Natufian, Neolithic and Chalcolithic (combined), Roman/Byzantine, and Modern populations for form and shape using the set of nine landmarks.

Population		Form (p*)	Shape (p*)
Natufian	Neolithic & Chalcolithic	0.054	**0**.**006**
	Roman/Byzantine	0.156	**0**.**006**
	Modern	**0**.**012**	**0**.**006**
Neolithic & Chalcolithic	Roman/Byzantine	1.000	1.000
	Modern	**0**.**012**	**0**.**036**
Roman/Byzantine	Modern	**0**.**018**	**0**.**024**

*Bonferroni-corrected *p* values.

**Table 2:** Linear discriminant analysis for determining the correct classification rate of populations according to hemimandibular form or shape after cross-validation (the Jackknife method).

Population	Correct classification rate (%)	
	Form	Shape
Pre-agriculture (Natufian)	70.0	80.0
Post-agriculture (Neolithic, Chalcolithic & Roman/Byzantine)	67.7	71.0
Post-industrial (Modern)	73.7	80.7
Total	71.4	77.6

### Shape analysis

**Figure 3:**
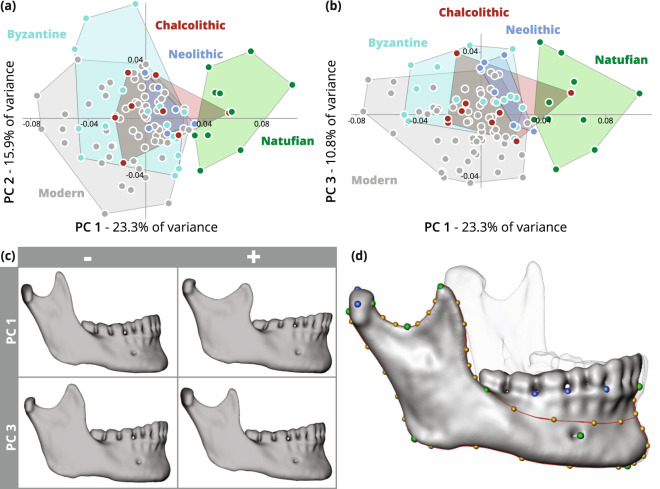
Principal component analysis of hemimandibular shape by population (using the set of nine landmarks): Natufian (green), Neolithic (blue), Chalcolithic (brown), Roman/Byzantine (light blue) and modern (gray). (**a**) The first two principal components (PCs) explain 39.2% of the variance. (**b**) The first and third PCs explain 34.1% of the variance . (**c**) Warped surfaces of the extreme values received for the first and third principal components (PC) using the set of 52 landmarks and semilandmarks (Supplementary Figure 1). Changes throughout the first PC, from the pre-agricultural population (the Natufian) to the post-industrial population (the Modern), appear in the mandibular body, which becomes more triangular (due to a reduction in the height of the posterior part of the mandibular body relative to the anterior part); the mandibular ramus, which becomes more narrow, elongated, and posteriorly tilted; the coronoid process, which becomes more narrow and elongated, extending beyond the condyle height; and the mandibular notch, which becomes narrower and deeper. Changes in shape throughout the third PC from post-Natufian (Neolithic, Chalcolithic, and Roman/Byzantine) to post-industrial populations exhibit an increase in chin projection, a narrowing of the coronoid, and a lengthening of the condyle. (**d**) Geometric morphometrics models used in the study: position of landmarks in the nine-landmark set marked with green dots. The position of landmarks in the fifty-two-landmark set marked with green and blue dots, and semilandmarks with yellow dots. Curves are marked with red lines.

**Figure 4:**
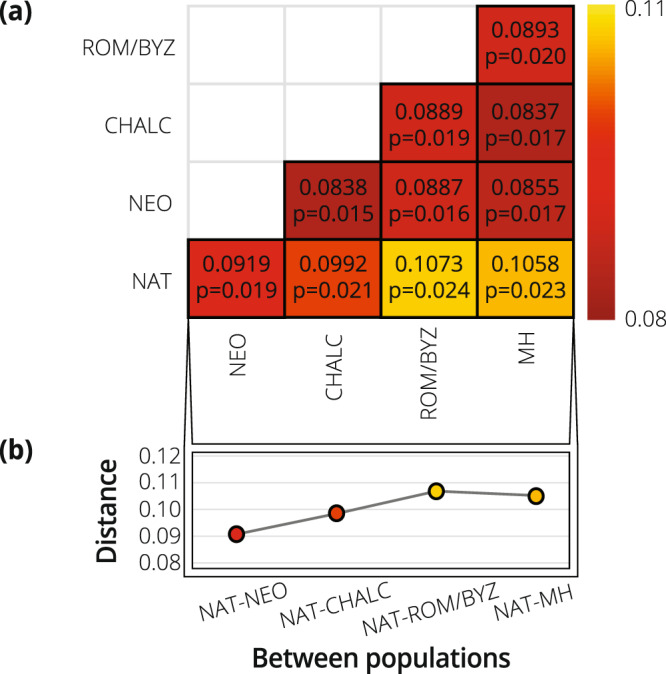
Mean and standard deviation of Procrustes distances between populations: (**a**) Colored matrix of Procrustes distances; dark red denotes the smallest mean distance, yellow the largest mean of Procrustes distances. (**b**) Mean of Procrustes differences between the Natufian and each of the studied populations. A significant gradual increase is presented until the Roman/Byzantine period. Statistical results appear in Table 3.

The first three components of the PCA explain 50% of the shape variance (Fig. 3a,b). The first PC, which explains 23.3% of the variance, distinguishes between the shape of the Natufian hemimandibles and the other hemimandibles. The shape variance of the Neolithic hemimandibles falls within the variance of the shape of the Chalcolithic hemimandibles, which is spread around the center of the Cartesian coordinate system, whereas the Roman/Byzantine and Modern hemimandibles exhibited a larger variance (Fig. 3a,b). The second PC, which explains 15.9% of the variance, differentiate slightly between the post-Natufian and Modern hemimandibles (Fig. 3a). The third PC, which explains 10.8% of the shape variance, distinguishes better the post-Natufian hemimandibles from the Modern ones (Fig. 3b). Since the Neolithic and Chalcolithic mandibles overlap entirely in the PCs, we combined them into one group for the pairwise PERMANOVA tests. The Natufian shape was significantly different from the other studied populations as well as the modern population (Table 1). The mean of Procrustes distances between populations increased significantly from the Natufian until the Roman/Byzantine period, and thereafter remained stable (Table 3; Fig. 4).

**Table 3:** One-Way ANOVA test (*p* < 0.05) with Tukey post hoc tests for examining differences in the mean Procrustes distances of shape variables between populations (based on the set of nine landmarks).

Distance between populations		*p*
Natufian-Neolithic	Natufian-Chalcolithic	0.219
	Natufian-Roman/Byzantine	**<0**.**01**
	Natufian-Modern	<**0**.**01**
Natufian-Chalcolithic	Natufian-Roman/Byzantine	**0**.**036**
	Natufian-Modern	0.054
Natufian-Roman/Byzantine	Natufian-Modern	0.880
Neolithic-Natufian	Neolithic-Chalcolithic	0.474
	Neolithic-Roman/Byzantine	0.993
	Neolithic-Modern	0.387
Neolithic-Chalcolithic	Neolithic-Roman/Byzantine	0.914
	Neolithic-Modern	1.000
Neolithic-Roman/Byzantine	Neolithic-Modern	0.933
Chalcolithic-Natufian	Chalcolithic-Neolithic	<**0**.**01**
	Chalcolithic-Roman/Byzantine	**0**.**010**
	Chalcolithic-Modern	<**0**.**01**
Chalcolithic-Neolithic	Chalcolithic-Roman/Byzantine	0.847
	Chalcolithic-Modern	1.000
Chalcolithic-Roman/Byzantine	Chalcolithic-Modern	0.149
Roman/Byzantine-Natufian	Roman/Byzantine-Neolithic	<**0**.**01**
	Roman/Byzantine-Chalcolithic	<**0**.**01**
	Roman/Byzantine-Modern	<**0**.**01**
Roman/Byzantine-Neolithic	Roman/Byzantine-Chalcolithic	1.000
	Roman/Byzantine-Modern	1.000
Roman/Byzantine-Chalcolithic	Roman/Byzantine-Modern	1.000
Modern-Natufian	Modern-Neolithic	<**0**.**01**
	Modern-Chalcolithic	<**0**.**01**
	Modern-Roman/Byzantine	<**0**.**01**
Modern-Neolithic	Modern-Chalcolithic	0.954
	Modern-Roman/Byzantine	0.079
Modern-Chalcolithic	Modern-Roman/Byzantine	<**0**.**01**

Since no significant differences in shape were found between the post-Natufian populations, we combined them for the LDA. Accordingly, the hemimandibular shape provides a correct classification rate, after cross-validation (using the Jackknife method), of 80% for the Natufian and modern populations and 71% for the post-Natufian populations (Table 2).

Since the 9 landmarks and 52 landmark and semilandmark sets produced similar results (see Supplementary Fig. 1 and Tables S2–S4), to improve the visualization of shape changes along the PC axes, the set of 52 landmarks and semilandmarks (see the Methods section) was used for surface warpings. Temporal shape changes along the first PC involved the mandibular body, i.e., from quadrangular to triangular shape (due to a reduction in the height of the posterior part of the mandibular body relative to the anterior part); the mandibular ramus, which becomes more narrow, elongated and posteriorly tilted; the coronoid process, which becomes more narrow and elongated, extending beyond the condyle height; and the mandibular notch, which becomes narrower and deeper (Fig. 3c). Changes in shape along the third PC suggest an increase in chin projection, a narrowing of the coronoid, and a lengthening of the condyle (Fig. 3c).

**Figure 5:**
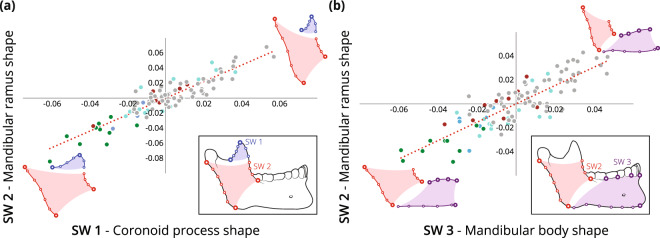
Plot of singular warps (SW) of coronoid process shape against mandibular ramus shape (**a**) and mandibular body shape against ramus shape (**b**), by population: Natufian (green), Neolithic (blue), Chalcolithic (brown), Roman/Byzantine (light blue), and modern (gray). Scores on these axes are significantly correlated (r = 0.911, p < 0.001, and r = 0.851, p < 0.001, respectively). Three-dimensional shape changes were presented schematically from the lateral view. The warpings of each block from the mean specimen shape towards the axis extremes were visualized utilizing PAST software (v. 3.15)^71^.

Associations between the shape of different regions of the mandible were examined via 2-block partial least square (PLS) analyses. The scores of individuals on the resulting first axes (singular warps, SW) of the shape of the mandibular ramus and coronoid, which explain 73.3% of the total covariance among blocks, show a correlation of 0.911 (*p* < 0.001), and the scores of individuals on the resulting first axes (SW) of the shape of the mandibular ramus and body, which explain 49.4% of the total covariance among blocks, show a correlation of 0.851 (*p* < 0.001) (Fig. 5). In both cases, the distribution along these axes reflects changes in mandibular shape over time, since the Natufian group is on one extreme and the modern mandibles are on the other one. In both cases, the Natufian mandibles can be differentiated from the other groups, whereas the Neolithic and Chalcolithic groups as well as the Roman/Byzantine and modern groups overlap considerably (Fig. 5).

## Discussion

The results of the current study suggest that the mandibles of the Levantine populations underwent considerable shape changes during the Holocene. A trend of size reduction was observed only from the hunter-gathering populations to the farming ones. The hypotheses raised in this study were confirmed, namely, that the shape of the mandible of the pre-agricultural population (the Natufians) differs from that observed in all succeeding populations of the Levant; the modern post-industrial population manifests a mandibular shape that is significantly different from all pre-industrial populations; and differences in mandibular shape increased over time.

The Natufian mandibles exhibit a short and wide ramus, a short and wide coronoid process, a wide mandibular notch, and a rectangular mandibular body. Modern mandibles manifest a long and narrow ramus that is tilted posteriorly, a long and narrow coronoid process, a narrow mandibular notch, a triangular mandibular body, and a projected chin. Modifications in mandibular shape throughout the Holocene were also reported for other populations and were generally attributed to changes in dietary habits, i.e., the transition to a less abrasive subsistence^1–3,33^. The major shape changes reported previously included the narrowing and elongation of the ramus and coronoid, the more obtuse mandibular angle, a shortening of the condyle, and a more projecting chin. Although all these studies support the masticatory-functional hypothesis, the changes in the mandibular size and shape do not fully overlap between the studies. This is probably because food abrasiveness is geographically dependent, hence, its different impact on mandibular shape^44^. For example, Galland *et al*.^3^, who studied three Nubian dietary groups (hunter-gatherers, early farmers, and late farmers) found trends of decreasing size and robusticity throughout the transition from hunting-gathering to farming: Mesolithic Nubians have a shorter, wider, and more upright ramus and coronoid process, a longer mandibular condyle, and a deeper, wider, and upright corpus. Katz *et al*.^2^ included in their “directional diet effects” a taller mandibular coronoid process, a narrower mandibular ramus, a more projecting lower chin, and a mandibular size reduction. Our study yielded similar results; nevertheless, it allowed us to follow changes in mandibular shape during the entire Holocene, including modern populations, in a restricted geographical region, as well as to locate subtle modifications in the mandible thanks to shape visualization based on a large battery of landmarks and semilandmarks.

The assumption that the mandibular morphological changes observed by others and us are related to subsistence strategy is strengthened by five unrelated findings. First, biomechanical and finite element studies demonstrated that mandibular shape is affected by the masticatory forces’ orientation and magnitude^33,45–49^. When loadings on mandibles (or their surrogates) are greater, the mandibular angle becomes narrower, the ramus and coronoid process becomes wider and shorter, and the mandibular body develops a rectangular shape^45,46^. Moreover, Stansfield and colleagues^33^ have suggested that differences in mandibular shape between populations is actually due to underdevelopment of the mandibles following reduced mastication loads during childhood, which is dependent on the subsistence strategy. Thus, mandibular shape can be used as an indicator of mastication loadings rather than of specific dietary inferences. Second, the changes in mandibular morphology occurred concomitantly with changes in dietary habits and food preparation techniques in Levantine Holocene populations. For example, the appearance of cookware in the Neolithic period or the introduction of dairy products in the Chalcolithic period^18–20,23,24^ reduced the biomechanical demands on the masticatory system^2^. Third, the association between food consistency and mandibular morphology has been confirmed by many experimental studies on animals^50–56^. Feeding rats and mice on soft and hard diets, for example, resulted in changes in ramus size and mandibular angle^51–54^. Similarly, pigs raised on a soft diet exhibited changes in jaw morphology and dental arch dimensions^55^, and hyraxes exposed to softer food exhibited shorter and lower mandibular bodies^56^. Fourth, there is other physiological evidence suggesting that the Natufian hunter-gatherers were subjected to greater mastication loadings compared with the succeeding populations, namely, the Natufians had broader and shorter faces^42,57^, which are considered to be characteristics of populations exposed to large mastication forces^58–63^. Five, no correlation between genetic factors and mandibular morphology was found^1^. In addition, ancient DNA studies of the Levantine populations found that whereas the Natufian and Neolithic populations have a similar local origin^64^, the Chalcolithic population (from the same archeological site that was studied here) are of a complex origin and are composed of 57% local Levant Neolithic, ~17% Iran Chalcolithic, and ~26% Anatolian Neolithic^65^. Nevertheless, the mandibular shape of the Neolithic mandible is more similar to that of the Chalcolithic rather than to the Natufian mandibles.

## Study Limitations

Since the sample sizes of the prehistoric and historic groups analyzed in the current study are relatively small, ranging from n = 6 to n = 16, the mean shape of each group is not representative. Therefore, in the current study we focused on shape variance, which is more precise^66^. In addition, the notion that changes in mandibular shape following the transition from hunting-gathering to farming is the primary factor behind the increase in orthodontic problems nowadays (for example, crowding and rotation)^1,6^ could not be tested in our study, since the sample size was too small and many teeth were lost postmortem.

## Conclusions

Considerable changes in mandibular shape are evident throughout the Holocene Levant. These changes can be explained by a reduction in the mastication forces applied on the mandible due to changes in the subsistence and food-producing techniques.

## Materials and Methods

The study included mandibles and hemimandibles of four prehistoric and historic populations of the Levant, samples of which are housed in the Anthropological Collection at the Sackler Faculty of Medicine, Tel Aviv University. The earliest population included 10 mandibles of hunter-gatherers (Natufians) (14,900–12,000 cal BP), followed by six mandibles of early farmers (Pre-pottery Neolithic C, PPNC) (7,400–6,150 cal BP), nine mandibles of Chalcolithic herders/farmers (6,500–5,800 cal BP), and 16 mandibles of individuals from the Roman-Byzantine period (see Supplementary Table S5 for further information about the mandibles included in the study). All mandibles underwent a high-resolution computerized tomography (CT) scan (Brilliance 64, Philips Medical Systems, Cleveland, Ohio: slice thickness 0.5–0.8 mm, 100 kV, 150 mAs, rotation time 0.75 sec, pitch 0.39, and Matrix 768*768) at the Carmel Medical Center, Haifa, Israel or were surface scanned via a Space Spider portable 3D scanner (Artec Europe, Luxembourg) at the Department of Anatomy and Anthropology, Sackler Faculty of Medicine, Tel Aviv University. Mandibles exhibiting pathological conditions (e.g., trauma or periodontal diseases) were excluded from the study. A modern sample was also included in the study and consisted of 62 mandibles (30 males and 32 females) of individuals aged 20–45 years who underwent a head CT scan between the years 2000 and 2012 (Brilliance 64, Philips Medical System, Cleveland, Ohio: slice thickness 0.9–3.0 mm, pixel spacing 0.3–0.5 mm, 120 kV, 250–500 mAs, number of slices 150–950 and Matrix 512*512) for medical purposes, at Carmel Medical Center, Haifa, Israel (approved by the ethics board of the Carmel Medical Center, number: 0066-11-CMC). Inclusion criteria of these mandibles included intact lower incisors and at least two teeth of the posterior unit (premolars and/or molars) on each side. Exclusion criteria included the absence of the lower incisors; dental implants and metal restorations that interfere with imaging and consequently, measurement; prominent facial and mandibular asymmetry; a craniofacial, temporomandibular joint or muscular disorders; trauma; previous surgery on the head and neck region (based on medical files or signs on the skull); and technically aberrant CT scans. In addition, outliers were removed from the analysis following Cardini *et al*.^67^. For mandibles that were scanned via CT, a 3D surface mesh of the mandibles was reconstructed from CT stacks using Amira 6.3 software (www.fei.com). A semi-automated segmentation of CT stacks was carried out based on gray level thresholds. Manual segmentation was performed where needed. For those scanned by the surface scanner, image processing and alignment were carried out via Artec Studio 13 software (Artec Europe, Luxembourg). The 3D shape of the mandible was analyzed using the Procrustes-based geometric morphometrics method. Since the sample size of the prehistoric and historic populations is limited, we carried out form and shape analyses on hemimandibles only, to maintain an appropriate p to n ratio^68^. The default side to be included in the study was right unless it was fractured or missing, in which case the left side was included, and landmarks were mirrored (Supplementary Table S5). The centroid size (henceforth, named size) for each hemimandible was calculated from landmarks, i.e., the square root of the sum of squared distances of landmarks from the centroid.

Two sets of landmarks were used in this study. The first included nine landmarks (Table 4, Fig. 3d). The number of landmarks to be included in the study was calculated according to Bookstein’s^69^ recommendations for maintaining a reasonable p to n ratio for revealing real shape differences. The second set includes 52 landmarks and semilandmarks and captures more details regarding the shape of the hemimandible (Tables 4 and 5, Fig. 3d). This set included 16 landmarks and 36 semilandmarks (representing 8 curves) and is based on a protocol already published by Sella Tunis *et al*.^45^. The second set of landmarks was used for visualization only. In both sets, landmarks were placed by the same researcher on the 3D surface mesh of the mandible using Evan Toolbox software 1.72 (www.evan-society.org). Semilandmark sliding was carried out based on the minimum bending energy technique^70^.

**Table 4:** Landmarks definitions (by set) for the landmarks used in the study.

Landmark		Definition	Landmarks used	
			52 set	9 set
1	Gnathion	The inferiormost point of the mandibular body in the midsagittal plane	V	V
2	Infradentale anterior	The anteriormost point of the mandibular alveolar border in the midsagittal plane	V	V
3	C-P3	The anteriormost point between the canine and the 1^st^ premolar (right)	V	
4	P4-M1	The anteriormost point between the 2^nd^ premolar and the 1^st^ molar (right)	V	
5	M1-M2	The anteriormost point between the 1^st^ and 2^nd^ molars (right)	V	
6	Mental foramen	The anteriormost point of mental foramen (right)	V	V
7	Root of ramus	The anteriormost point of the ramus rim at the level of the alveolar ridge (right)	V	V
8	Gonion	The point on the projection of the bisection of the mandibular angle (right)	V	V
9	Lateral condyle	From a superior view, the lateralmost point of the condyle (right)	V	
10	Center of condyle	From a superior view, the central point of the condyle (right)	V	
11	Sigmoid notch	The inferiormost point of the mandibular notch, when the mandible is positioned in the mandibular plane (right)	V	V
12	Coronion	The superiormost point of the coronoid process (right)	V	V
13	Mandibular foramen	The inferiormost point of the mandibular foramen (right)	V	
14	Alveolar process - lingual aspect	From a superior view, the intersection between a line tangent to the lingual alveolar process of the molar teeth and a line perpendicular to it, passing through the ramus root (right)	V	
15	Anterior condyle	The anterosuperior point of the mandibular notch (right)	V	V
16	Posterior condyle	The posteriormost point of the condyle at its center (right)	V	V

**Table 5:** Definition of curves and the number of semilandmarks (sLMs) placed on each curve in the 52 set of landmarks and semilandmarks.

Curve		Definition	# of sLMs
1	Mandibular body (right)	Passing from the root of ramus (LM 7) along an oblique line to the midheight of the mandibular symphysis	4
2	Anterior rim of ramus (right)	Passing from coronion (LM 12) to the root of ramus (LM 7)	5
3	Inferior margin of the mandibular body (right)	Passing from gonion (LM 8) to gnathion (LM 1)	5
4	Posterior rim of ramus (right)	Passing from posterior condyle (LM 16) to gonion (LM 8)	5
5	Mandibular notch	Passing from anterior condyle (LM 15) to coronion (LM 12) on the superior border of the mandibular notch	5
6	Anterior symphysis	Passing from infradentale (LM 2) to the anteriormost point in the midsagittal plane	3
7	Inferior symphysis	Passing from the anteriormost point in the midsagittal plane to the genial tubercle	6
8	Posterior symphysis	Passing from the genial tubercle to the postero-superior point of the mandibular alveolar border in the midsagittal plane	3

### Statistical analyses

Data were analyzed using PAST software (v. 3.15)^71^. Significance was set at *p* < 0.05. Cartesian coordinates were converted into form or shape variables using General Procrustes Analysis (GPA). Outliers were examined for each population based on 95% confidence intervals^67^. Since mandibular size affects shape variation^44,72,73^, we controlled for allometry using the accepted method of multivariate regression of shape variables onto the centroid size (allometrically adjusted)^66,70^ via the EVAN Toolbox 1.72 (www.evan-society.org). Most cases of hemimandibles were of the right side (Supplementary Table S5); in those where only the left side was available, we used the mirrored landmarks and semilandmarks (converted in EVAN Toolbox 1.72). PCA was carried out to examine the form and shape variance in the studied populations. Pairwise permutational multivariate analyses of variance (PERMANOVA) with 1000 permutations and Bonferroni-corrected *p* values were carried out to examine differences between groups based on Procrustes shape distances in Euclidean space. Kruskal-Wallis test with post hoc pairwise comparisons (Mann-Whitney tests with Bonferroni corrections) were carried out to examine differences in size between the studied groups. One-Way ANOVA with Tukey Post Hoc tests were carried out to examine differences in the mean Procrustes distances between groups. LDA with cross-validation (using the Jackknife method) was carried out to estimate the correct classification rate of groups according to their mandibular form and shape. Two-block PLS analyses were carried out to examine correlations between the shape of different mandibular regions. Shape changes were visualized by warping the mean surface mesh using a triplet of thin plate splines (TPS)^74^ in the EVAN Toolbox 1.72 (www.evan-society.org).

Intra- and inter-observer variations for the GM protocol were tested and published previously^45^.

## Supplementary information

Supplementary Information

## Data Availability

The datasets analyzed during the current study are available from the corresponding author upon request.
