# Supplementary material for: Changes in human mandibular shape during the Terminal Pleistocene-Holocene Levant
Source: Sci Rep. 2019 Jun 19;9:8799. doi: 10.1038/s41598-019-45279-9 (PMC6584575; doi:10.1038/s41598-019-45279-9)
Supplement: Supplementary file 1 — Supplementary Information [file 41598_2019_45279_MOESM1_ESM.docx]

**Supplementary Information:**

**Changes in human mandibular shape during the Terminal Pleistocene-Holocene Levant**

Ariel Pokhojaev^1-3^, Hadas Leah Levine ^1,2^, Tatiana Sella-Tunis^1-3^, Rachel Sarig^2,3^, and Hila May^1,2*^

^1^ Department of Anatomy and Anthropology, Sackler Faculty of Medicine, Tel Aviv University, Ramat Aviv, Tel Aviv 69978, Israel

^2^ Shmunis Family Anthropology Institute, the Dan David Center for Human Evolution and Biohistory Research, The Steinhardt Museum of Natural History, Sackler Faculty of Medicine, Tel Aviv University, Ramat Aviv, Tel Aviv 69978, Israel

^3^ Departments of Orthodontics and Oral Biology, The Maurice and Gabriela Goldschleger School of Dental Medicine, Sackler Faculty of Medicine, Tel Aviv University, Ramat Aviv, Tel Aviv 69978, Israel

**
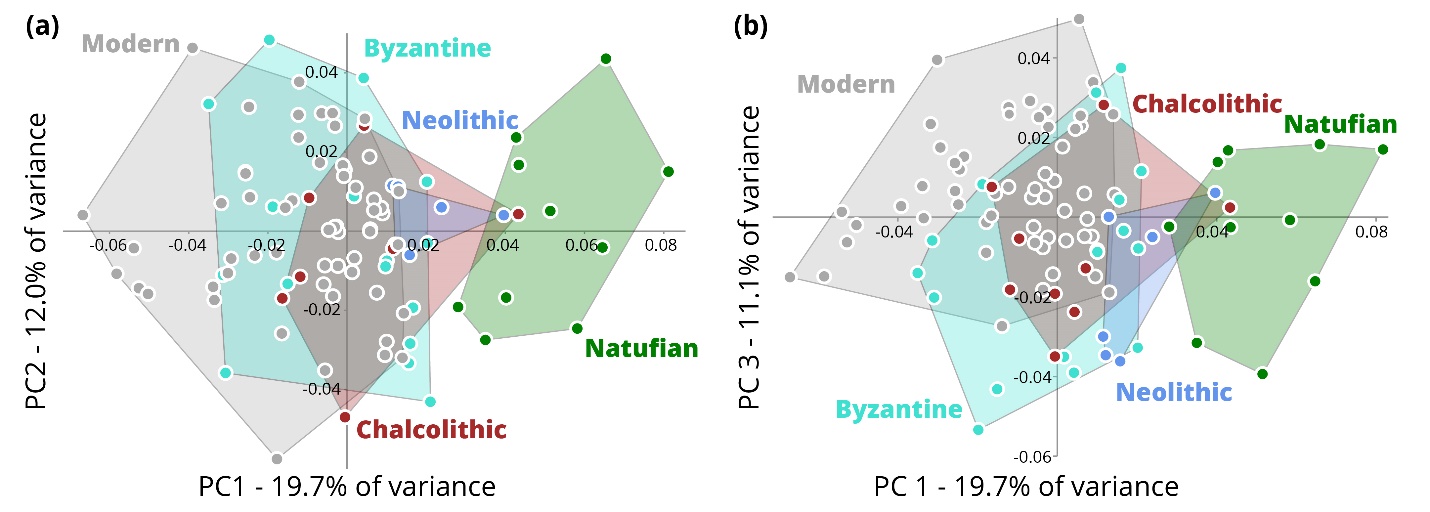
**

**Supplementary Figure S1:** Principal component analysis of hemimandibular shape by population using the set of 52 landmarks and semilandmarks. Natufian (green), Neolithic (blue), Chalcolithic (brown), Roman/Byzantine (light blue), and modern (gray)

**Supplementary Table S1:** Descriptive statistics and Kruskal-Wallis test with post hoc pairwise comparisons (Mann-Whitney tests with Bonferroni corrections) for examining size differences between the Natufian, Neolithic, Chalcolithic, Roman/Byzantine and Modern hemimandibles.

| **Population** | **Mean (±SD)** | **Population** | *p* |
| --- | --- | --- | --- |
| Natufian | 138.7 (±8.98) | Neolithic | 0.927 |
|  |  | Chalcolithic | 1 |
|  |  | Roman/Byzantine | 1 |
|  |  | Modern | 1 |
| Neolithic | 130.9 (±3.69) | Chalcolithic | 1 |
|  |  | Roman/Byzantine | 1 |
|  |  | Modern | 0.118 |
| Chalcolithic | 134.4 (±6.92) | Roman/Byzantine | 1 |
|  |  | Modern | 1 |
| Roman/Byzantine | 134 (±9.90) | Modern | 0.200 |
| Modern | 140 (±8.30) |  | |

**Supplementary Table S2:** Pairwise permutational multivariate analysis of variance (with 1000 permutations) on Procrustes distances between Natufian, Neolithic, and Chalcolithic (combined), Roman/Byzantine, and Modern populations using the set of 52 landmarks and semilandmarks.

| **Population** | | **Form (*p*^*^)** | **Shape (*p*^*^)** |
| --- | --- | --- | --- |
| Natufian | Neolithic & Chalcolithic | **0.036** | **0.006** |
|  | Roman/Byzantine | 0.228 | **0.006** |
|  | Modern | **0.006** | **0.006** |
| Neolithic & Chalcolithic | Roman/Byzantine | 1.000 | 1.000 |
|  | Modern | **0.012** | **0.006** |
| Roman/Byzantine | Modern | **0.060** | **0.006** |

^*^Bonferroni-corrected *p* values

**Supplementary Table S3:** Descriptive statistics (mean and standard deviation) of Procrustes distances between every two populations that were included in the study (based on the set of 52 landmarks and semilandmarks).

| **Distance between populations** | **Mean (±SD)** |
| --- | --- |
| Natufian-Neolithic | 0.085 (±0.014) |
| Natufian-Chalcolithic | 0.092 (±0.015) |
| Natufian-Roman/Byzantine | 0.098 (±0.017) |
| Natufian-Modern | 0.099 (±0.017) |
| Neolithic-Chalcolithic | 0.082(±0.014) |
| Neolithic-Roman/Byzantine | 0.085 (±0.013) |
| Neolithic-Modern | 0.085 (±0.013) |
| Chalcolithic-Roman/Byzantine | 0.084 (±0.014) |
| Chalcolithic-Modern | 0.081 (±0.014) |
| Roman/Byzantine-Modern | 0.086 (±0.015) |

**Supplementary Table S4:** One-Way ANOVA test (*p*<0.05*)* with Tukey Post Hoc tests for examining differences in mean Procrustes distances between populations (based on the set of 52 landmarks and semilandmarks).

| **Distance between populations** | | | ***p*** | |
| --- | --- | --- | --- | --- |
| Natufian-Neolithic | Natufian-Chalcolithic | 0.123 | |  |
|  | Natufian-Roman/Byzantine | <0.01 | |  |
|  | Natufian-Modern | <0.01 | |  |
| Natufian-Chalcolithic | Natufian-Roman/Byzantine | 0.163 | |  |
|  | Natufian-Modern | 0.007 | |  |
| Natufian-Roman/Byzantine | Natufian-Modern | 0.999 | |  |
| Neolithic-Natufian | Neolithic-Chalcolithic | 0.990 | |  |
|  | Neolithic-Roman/Byzantine | 1.000 | |  |
|  | Neolithic-Modern | 1.000 | |  |
| Neolithic-Chalcolithic | Neolithic-Roman/Byzantine | 0.951 | |  |
|  | Neolithic-Modern | 0.942 | |  |
| Neolithic-Roman/Byzantine | Neolithic-Modern | 1.000 | |  |
| Chalcolithic-Natufian | Chalcolithic-Neolithic | 0.004 | |  |
|  | Chalcolithic-Roman/Byzantine | 0.003 | |  |
|  | Chalcolithic-Modern | <0.01 | |  |
| Chalcolithic-Neolithic | Chalcolithic-Roman/Byzantine | 0.998 | |  |
|  | Chalcolithic-Modern | 1.000 | |  |
| Chalcolithic-Roman/Byzantine | Chalcolithic-Modern | 0.698 | |  |
| Roman/Byzantine-Natufian | Roman/Byzantine-Neolithic | <0.01 | |  |
|  | Roman/Byzantine-Chalcolithic | <0.01 | |  |
|  | Roman/Byzantine-Modern | <0.01 | |  |
| Roman/Byzantine-Neolithic | Roman/Byzantine-Chalcolithic | 1.000 | |  |
|  | Roman/Byzantine-Modern | 1.000 | |  |
| Roman/Byzantine-Chalcolithic | Roman/Byzantine-Modern | 0.961 | |  |
| Modern-Natufian | Modern-Neolithic | <0.01 | |  |
|  | Modern-Chalcolithic | <0.01 | |  |
|  | Modern-Roman/Byzantine | <0.01 | |  |
| Modern-Neolithic | Modern-Chalcolithic | 0.025 | |  |
|  | Modern-Roman/Byzantine | 0.998 | |  |
| Modern-Chalcolithic | Modern-Roman/Byzantine | <0.01 | |  |

**Supplementary Table S5:** Information regarding prehistoric and historic samples included in the study

| **Period** | **Site & Sample size (N)** | **Specimen ID** | **Sex** | **Age (yr)** | **Condition of mandible** | **Side included*** |
| --- | --- | --- | --- | --- | --- | --- |
| **Natufian**  12,500-11,830 cal BP | Eynan/ Mallaha  (N=5) | EM H91 | N/A | Adult^77^ | Partial right side; complete left side | Left |
|  |  | EM H80 |  |  | Missing right coronion and left condyle^#^ | Right |
|  |  | EM H37 |  |  | Partial right side; complete left side | Left |
|  |  | EM H23 |  |  | Left hemi-mandible | Left |
|  |  | EM H18 |  |  | Right hemi-mandible | Right |
|  | Hayonim^75^  (N=5) | HAY GVIII H20 | M | 25-30 | Complete right side; partial left side | Right |
|  |  | HAY GIX H25 | M | ~25 | Right hemi-mandible | Right |
|  |  | HAY GIX H27 | M | 35-45 | Partial right side; complete left side | Left |
|  |  | HAY GXI H20 | N/A | Adult | Complete right side; partial left side | Right |
|  |  | HAY GXI H29 | M | 17-18 | Partial right side; complete left side | Left |
| **Prepottery Neolithic C**  8,900-8,350 cal BP | Atlit Yam^76^  (N=6) | AY H1 | F | Adult^77^ | Right hemi-mandible | Right |
|  |  | AY H5 | N/A |  | Partial right side; complete left side | Left |
|  |  | AY H25 |  |  | Complete right side; partial left side | Right |
|  |  | AY H69 |  |  | Complete | Right |
|  |  | AY H91 |  |  | Partial right side; complete left side | Left |
| **Chalcolithic**  6,500–5,800 cal BP | Peki'in  (N=9) | CHPK37 | N/A | Adult^77^ | Complete | Right |
|  |  | CHPK18 |  |  | Partial right side; complete left side | Left |
|  |  | CHPK15 |  |  | Complete | Right |
|  |  | CHPK 23 |  |  | Partial right side; complete left side | Left |
|  |  | CHPK27 |  |  | Complete right side; partial left side | Right |
|  |  | CHPK28 |  |  | Complete | Right |
|  |  | CHPK31 |  |  | Complete right side; partial left side | Right |
|  |  | CHPK35 |  |  | Partial right side; complete left side | Left |
|  |  | CHPK36 |  |  | Complete right side; partial left side | Right |
| **Roman/Byzantine** 37 BC-638 AD | Various sites (N=16) | RB1 | N/A | Adult^77^ | Complete | Right |
|  |  | RB2 |  |  | Complete right side; partial left side | Right |
|  |  | RB3 |  |  | Complete right side; partial left side | Right |
|  |  | RB4 |  |  | Complete right side; partial left side | Right |
|  |  | RB5 |  |  | Partial right side; complete left side | Left |
|  |  | RB6 |  |  | Complete | Right |
|  |  | RB7 |  |  | Partial right side; complete left side | Left |
|  |  | RB8 |  |  | Partial right side; complete left side | Left |
|  |  | RB9 |  |  | Left hemi-mandible | Right |
|  |  | RB10 |  |  | Partial right mandibular body; partial left ramus^#^ | Right |
|  |  | RB11 |  |  | Complete | Right |
|  |  | RB12 |  |  | Complete right side; partial left side | Right |
|  |  | RB13 |  |  | Complete | Right |
|  |  | RB14 |  |  | Right hemi-mandible | Right |
|  |  | RB15 |  |  | Partial right and left sides^#^ | Right |
|  |  | RB16 |  |  | Partial right mandibular body; complete left side | Left |

^*^Left hemi-mandibles were mirrored via Amira v. 6.3.

^#^Missing part was reconstructed based on the left side by mirroring (Amira v. 6.3).

**References:**

75. Belfer-Cohen, A. The Natufian graveyard in Hayonim cave. *Paléorient*, 297-308 (1988).

76. Galili, E., Gopher, A., Eshed, V. & Hershkovitz, I. Burial practices at the submerged pre-pottery Neolithic C site of Atlit-Yam, Northern Coast of Israel. *Bulletin of the American Schools of Oriental Research*, 1-19 (2005).

77. Buikstra, J. E., & Ubelaker, D. H. Standards for data collection from human skeletal remains. *Arkansas Archaeological Survey Research Series*, *44* (1994).
